# Trans^2^-CBCT: A Dual-Transformer Framework for Sparse-View CBCT Reconstruction

**DOI:** 10.3390/s26154710

**Published:** 2026-07-24

**Authors:** Minmin Yang, Yunhui Zhu, Huantao Ren, Senem Velipasalar

**Affiliations:** Department of Electrical Engineering and Computer Science, Syracuse University, Syracuse, NY 13244, USA; myang47@syr.edu (M.Y.); hren11@syr.edu (H.R.); svelipas@syr.edu (S.V.)

**Keywords:** Trans2-CBCT, cone-beam computed tomography, CBCT reconstruction, transunet, neighbor-aware point transformer, neighbor-aware attention

## Abstract

Cone-beam computed tomography (CBCT) with sparse projection views offers reduced radiation dose and faster scans but introduces severe streak artifacts and spatial coverage gaps. We address these challenges within a unified framework. First, we replace conventional UNet/ResNet encoders with TransUNet, a hybrid CNN–Transformer architecture that jointly models local details and long-range spatial context. It is adapted to CBCT reconstruction by concatenating multi-scale feature maps and introducing a lightweight attenuation-prediction head. Trans-CBCT outperforms the best baseline by 1.17 dB in PSNR and by 0.0163 in SSIM on LUNA16 with only six projection views. Second, we incorporate a neighbor-aware Point Transformer with explicit 3D positional encodings and a neighbor-aware attention module aggregating information from each point’s *k*-nearest spatial neighbors to enforce volumetric coherence. The resulting Trans^2^-CBCT achieves an additional 0.63 dB increase in PSNR and 0.0117 increase in SSIM over Trans-CBCT. In experiments with 6-10 views, Trans-CBCT and Trans^2^-CBCT consistently outperform all prior methods in both PSNR and SSIM on LUNA16. On the ToothFairy dataset, Trans^2^-CBCT leads in five of the six measurements, outperforming all baselines in PSNR. These results highlight the effectiveness of combining hybrid CNN–Transformer features with geometry-aware point-based reasoning for sparse-view CBCT reconstruction.

## 1. Introduction

Early X-ray computed tomography (CT) systems relied on a narrow fan-beam geometry: A one-dimensional detector array collected projections as the X-ray source and detector rotated synchronously around a stationary patient, producing a stack of 2D sinograms that were reconstructed slice-by-slice. Decades of progress, spanning filtered backprojection algorithms, iterative statistical reconstruction methods [1,2,3], and advancements in applying different regularization terms [4,5], have steadily improved spatial resolution, noise performance, and scan speed. Cone-beam CT (CBCT) extends this evolution by replacing the fan beam with a cone-shaped X-ray beam and a two-dimensional flat-panel detector. A single gantry rotation now captures an entire volume instead of an individual slice, enabling faster acquisitions and isotropic voxels that are advantageous for dental, head-and-neck, and image-guided interventional applications. However, achieving diagnostic-quality reconstructions traditionally requires hundreds of projection views, leading to prolonged scan times and increased radiation exposure. These concerns have driven the development of sparse-view CBCT reconstruction.

Sparse-view CBCT reconstruction aims to reconstruct accurate three-dimensional volumes from a few projection views (typically six to ten views), thereby reducing the scanning time and lowering radiation dose. Learning-based methods have shown promise in addressing this challenge [6,7,8,9,10]. NeRF-inspired approaches [8,11] treat CBCT as a continuous attenuation field and optimize scan-specific parameters to minimize the discrepancy between real and synthesized projections. Although these methods are capable of capturing fine details, they demand intensive per-scan optimization and exhibit performance degradation when input views drop below ten. In contrast, data-driven methods [6,7,12] use deep neural networks, such as UNet [13] and ResNet [14] to extract features from sparse projections, then backproject these features into 3D space and refine 3D representations through MLPs or convolutional decoders. For instance, DIF-Net [6] utilizes a UNet encoder–decoder to extract image-level features, which are then processed by MLPs to predict voxel-wise intensities. Similarly, C^2^RV [7] employs UNet to learn multi-scale, multi-view features that are combined and aligned in multi-scale 3D volumetric representations. Another method [12] adopts ResNet34 [14] as the image encoder, projecting learned features into a 3D volume and reconstructing the final CBCT image using a 3D CNN decoder.

Despite their successes, a fundamental limitation of the UNet- or ResNet-based architectures is their inherent reliance on local convolutional operations, restricting their ability to effectively capture global context and long-range spatial dependencies. In this work, to overcome this bottleneck, we first replace UNet/ResNet with TransUNet [15], a hybrid CNN–Transformer, whose convolutions capture fine local details while its self-attention layers provide the global context. For each projection, we extract multi-scale TransUNet features, query view-specific features given 3D points, and fuse the 3D features across all views via max-pooling. The aggregated 3D features are then forwarded to a lightweight attenuation-prediction head, yielding our first proposed model, Trans-CBCT, which already surpasses the baselines, in terms of both signal-to-noise ratio (PSNR) [16] and structural similarity index measure (SSIM) [17], and improves performance on both the LUNA16 [18] and ToothFairy [19] datasets. Although Trans-CBCT delivers favorable results, the reconstructed volume can still be improved to be spatially more coherent. Hence, we also introduce a neighbor-aware Point Transformer to refine 3D features and improve spatial consistency. Sparse-view CBCT reconstruction often struggles with maintaining accurate spatial relationships given sparse projection views. The Point Transformer overcomes this challenge by adding 3D coordinate positional encodings to the 3D point features, which enables the network to reason about the spatial positioning of features more effectively. Moreover, we integrate a KNN-based neighbor-aware attention mechanism within the Point Transformer, which helps in preserving local details and promoting spatial smoothness. This mechanism selectively aggregates information from spatially neighboring points, thereby mitigating aliasing artifacts and ensuring that local anatomical details are not lost. Combining TransUNet’s global feature extraction with our neighbor-aware Point Transformer’s locality-aware refinement yields a unified framework that delivers state-of-the-art accuracy on sparse-view CBCT reconstruction.

To summarize, the main contributions of this work include the following:

- We replace the conventional UNet- or ResNet-based architectures with TransUNet, resulting in our first model, referred to as the Trans-CBCT. The task-specific design decisions are as follows: (a) repurposing the decoder’s intermediate activations as an externally queryable multi-scale feature bank for 3D point sampling, rather than as an internal pathway toward a single mask; (b) exposing and concatenating the activations from all four decoder stages as the per-point feature, rather than querying only the final decoder output; (c) embedding this encoder inside a project–interpolate–max-pool cross-view fusion pipeline that aligns 3D query points to per-view 2D features; and (d) replacing the segmentation head with a prediction head. The novelty here is not the use of multiple scales but the decision of which decoder stages to expose for cross-view point querying, together with the surrounding 3D reconstruction machinery, which is specific to our setting. By introducing Trans-CBCT, we demonstrate that these task-specific adaptations produce consistent, sizable gains in sparse-view CBCT reconstruction across two datasets.
- We introduce a neighbor-aware Point Transformer whose core mechanism restricts multi-head attention to each point’s nearest spatial neighbors, aggregating local neighbor features to reduce aliasing artifacts and preserve fine structures. We further enhance it with learnable 3D positional encodings and a Gaussian distance bias that incorporate explicit geometry into the attention, providing an additional refinement in spatial consistency. This results in our proposed Trans^2^-CBCT.
- By integrating TransUNet with coordinate-aware point reasoning and locality-driven attention, Trans^2^-CBCT achieves high-fidelity 3D reconstructions from extremely sparse (6–10 views) CBCT data, outperforming the existing baselines and providing state-of-the-art performance.

## 2. Related Work

### 2.1. Sparse-View CBCT Reconstruction

In sparse-view CBCT, the goal is to reconstruct volumetric images from significantly fewer X-ray projection angles than conventional scans, reducing radiation dose and scan time. However, angular undersampling leads to severe streak artifacts and loss of fine structures when using traditional analytic reconstruction techniques. This challenge has motivated a broad range of methods, including iterative and deep learning-based approaches, each seeking to balance volumetric image quality, computational efficiency, and radiation safety.

(i) Traditional and Iterative Methods

The Feldkamp–Davis–Kress (FDK) [20] algorithm performs a 1D weighted filtering of the projection data along detector rows, followed by approximate backprojection along divergent cone-beam paths. Despite its speed and simplicity, FDK produces pronounced streak artifacts and resolution loss under sparse-view sampling, because it lacks mechanisms to enforce consistency across views or incorporate prior information. To address these artifacts, iterative reconstruction methods formulate CBCT as an optimization problem, incorporating regularization terms to impose sparsity smoothness. The Algebraic Reconstruction Technique (ART) [1,21] and Simultaneous Algebraic Reconstruction Technique (SART) [2] iterate between forward and backward projections to progressively refine the image. Total variation (TV) minimization [5] and its variants [22,23] enforce sparsity of image gradients, effectively reducing streak artifacts in sparse-view data. These TV-based methods belong to the broader family of compressed-sensing-inspired (CSI) algorithms, which enable high-quality reconstruction from undersampled data [24]. Representative CSI algorithms include the algebraic reconstruction technique with TV-regularization and adaptive segmentation (ART-TVS) [25] and the iterative Potts minimization algorithm (IPMA) [26].

(ii) Deep Learning-based Methods

With the advances in deep neural networks, data-driven methods have achieved remarkable results. Deep Back Projection [27] feeds individual backprojected views into a CNN to learn a mapping from noisy backprojections to artifact-free images. FBPConvNet [28] applies a UNet to FBP-reconstructed images, training on paired sparse-view and full-view reconstruction. Recent implicit neural representation (INR) methods treat the attenuation field as a continuous function parameterized by a coordinate-based MLP. PixelNeRF [29] and its adaptations [8,30] leverage sparse views to condition neural field on projection data, enabling high-fidelity reconstructions under extreme sparse-view settings. Such INR approaches implicitly encode volumetric consistency and have shown promising results, particularly when combined with Gaussian representations [9,31]. DIF-Net [6] uses a 2D UNet to extract feature maps from each input view, projects arbitrary 3D query points into those maps to sample view-specific features, fuses them via an MLP, and regresses per-point intensities. DIF-Gaussian [31] also uses a UNet feature extractor but replaces dense voxels with 3D Gaussians to embed spatial priors, improving point-wise intensity prediction. C^2^RV [7] further extends UNet by extracting multi-scale 2D feature maps, backprojecting them into explicit 3D volumes for cross-regional learning, and using a scale-view cross-attention module to fuse voxel-aligned and pixel-aligned features. While all three methods [6,7,31] leverage UNet’s strong local feature encoding, they lack an explicit mechanism for modeling long-range spatial dependencies or global context across the entire image plane—an important factor when reconstructing from extremely sparse views. To address this, we replace the standard UNet encoder with TransUNet [15], which integrates a vision-transformer block into the bottleneck. This hybrid design preserves UNet’s high-resolution, multi-scale feature maps while adding self-attention layers that capture non-local correlations and global structure. By adopting TransUNet, our framework yields richer and more globally coherent image features that translate into more accurate CBCT reconstruction that is robust to artifacts under extreme sparsity.

### 2.2. Transformers in Medical Imaging

Transformer architectures, initially popularized for images by Vision Transformers (ViT) [32], have been increasingly adopted in medical imaging tasks, including classification and segmentation. Owing to their ability to model long-range dependencies, Transformers have emerged as powerful alternatives to traditional CNNs, especially when capturing complex global contexts is critical.

(i) Transformers for Medical Image Classification

Transformer architectures have been applied to different medical image modalities to achieve classification. TL-Med [33] employs a two-stage transfer learning strategy utilizing the ViT architecture. At the first stage, the ViT model is pretrained on the ImageNet [34] to capture generic features. Then, the model is fine-tuned on a tuberculosis CT scan dataset. At the second stage, the model is further fine-tuned on a COVID-19 CT scan dataset. This stage refines the model’s ability to distinguish between COVID-19 and non-COVID-19 cases. [35] introduces a Transformer into multi-modal medical image classification by modeling both spatial and modality-wise correlations through self-attention, achieving superior performance in handling missing or noisy modalities. Mohamed et al. [36] proposed the use of ViTs for breast ultrasound image classification, demonstrating that the self-attention mechanism enables more effective modeling of long-range dependencies compared to conventional CNNs. By directly processing flattened image patches, the ViT architecture better captures global anatomical patterns, leading to improved classification performance, particularly in distinguishing malignant from benign lesions.

(ii) Transformers for Medical Image Segmentation

Transformers have also shown promise in medical image segmentation, where capturing both local details and global anatomical context is crucial. Swin UNETR [37] leverages a Swin Transformer [38] as the encoder within a U-shaped architecture for brain tumor segmentation in MRI scans. By using hierarchical feature maps and local window-based self-attention, Swin UNETR significantly improves segmentation accuracy compared to CNN-based methods. VT-UNet [39] pushed this further by removing convolutional components entirely and by using ViT throughout the architecture. VT-UNet employs hierarchical volumetric self-attention with shifted windows, parallel self- and cross-attention in the decoder, and Fourier positional encoding, achieving good tumor segmentation accuracy and robustness with lower computational complexity. Meanwhile, specialized designs, such as D-Former [40], introduce a novel self-attention mechanism that alternates between local scope modules and global scope modules, and use 3D depth-wise convolutions to learn position information dynamically. With these modules, D-Former offers a good trade-off between accuracy and computational cost. TransUNet [15] combines CNN-based encoders with Transformer modules to simultaneously capture local fine-grained features and global context, significantly improving segmentation performance.

We adopt TransUNet as the 2D feature extractor for X-ray projection images due to its architectural simplicity and effectiveness in 2D medical image analysis. Unlike Swin UNETR and VT-UNet, which are primarily designed for 3D volumetric segmentation, TransUNet operates on 2D projection images and is more suitable for our task. Moreover, its modular CNN–Transformer structure allows us to easily extract and concatenate multi-scale features, which is crucial for our cross-view fusion and 3D point-wise prediction.

(iii) Transformers for CBCT

Recently, Transformer architecture has also been explored for improving CBCT images. For example, Cao et al. [41] proposed a 4D-CBCT method that leverages Swin Transformers [38] and masking strategies to improve temporal consistency and reconstruction robustness. Yuan et al. [42] introduced WUTrans, a whole-spectrum unilateral-query-secured Transformer tailored for 4D CBCT reconstruction, aiming to model both spatial and temporal dynamics with improved query efficiency. Zhao et al. [43] presented CBCTformer, a Transformer-based artifact reduction model designed to enhance already-reconstructed CBCT volumes. Sha et al. [44] further address ring artifact suppression using a Transformer model trained with a dual-domain loss, including a novel unidirectional vertical gradient loss in polar coordinates. Gao et al. [45] presented Dual-Swin, a dual-domain reconstruction framework that reconstructs a field-of-view (FOV) extended CBCT image from truncated sinograms by applying Swin Transformers in both sinogram and image domains. While effective for addressing truncation artifacts in radiation therapy, Dual-Swin assumes relatively dense angular sampling and focuses on sinogram restoration, whereas our work targets direct 3D reconstruction from extremely sparse projections using a unified dual-Transformer framework.

While the aforementioned works demonstrate the growing role of Transformers in CBCT-related tasks, they often rely on relatively dense projection data or operate in a post-reconstruction refinement setting. In contrast, our work targets the challenging problem of direct 3D CBCT reconstruction from an extremely sparse number of views. We introduce a unified dual-Transformer framework that adapts TransUNet for 2D projection-level feature extraction and introduces a neighbor-aware Point Transformer for 3D spatial reasoning, enabling high-fidelity volumetric reconstruction from as few as six X-ray projections.

In summary, prior methods fall into three families. Scan-specific INR methods, such as NAF [8] and NeRP [30], optimize a continuous attenuation field per scan using only the input projections, without learned priors from external data. They require costly per-scan optimization, and their performance drops once the number of views enters the extremely sparse regime. Data-driven methods such as DIF-Net [6], DIF-Gaussian [31], and C^2^RV [7] learn cross-scan priors and are more robust, but rely on local convolutional operations and lack an explicit mechanism for global context and long-range spatial dependencies across the projection plane. Existing transformer-based CBCT methods [41,42,43,44,45] introduce global modeling but rely on relatively dense projection data or operate in a post-reconstruction refinement setting, and thus cannot directly solve the 3D reconstruction problem from sparse projections. Across all three families, the 3D representation is typically refined without an explicit constraint enforcing local geometric coherence among neighboring voxels, leaving reconstructions prone to aliasing and loss of fine anatomical detail. Our proposed Trans^2^-CBCT addresses these limitations by using TransUNet to capture both local and global 2D features and a neighbor-aware Point Transformer that provides explicit 3D spatial reasoning under extreme sparsity.

## 3. Proposed Method

### 3.1. Revisit of TransUNet

**Figure 1:**
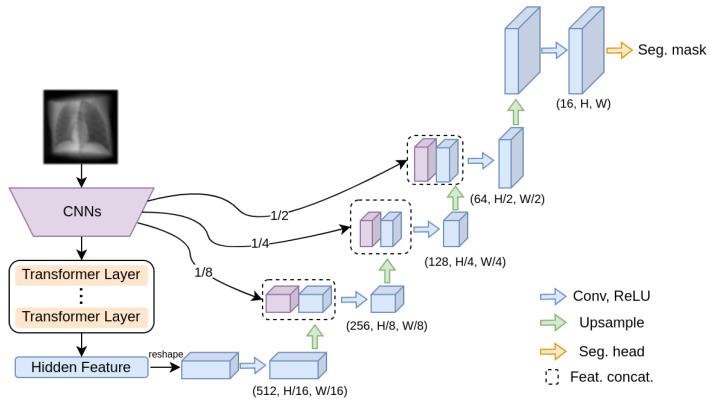
The architecture of TransUNet [15]. It includes a CNN–Transformer hybrid model as encoder and a cascaded upsampler to decode the hidden features.

TransUNet [15] significantly advanced medical image segmentation by combining the complementary strengths of convolutional neural networks (CNNs) and vision transformers. As illustrated in Figure 1, TransUNet employs a hybrid architecture, wherein an initial convolutional encoder captures hierarchical local features from the input images. These CNN-derived features are then processed by transformer layers, utilizing self-attention mechanisms to effectively capture long-range, global contextual relationships.

A notable innovation of TransUNet lies in its U-shaped structure, which integrates globally aware transformer representations with detailed CNN features through skip connections and upsampling operations. This design ensures that crucial fine-grained details are preserved while simultaneously benefiting from the transformer’s global context modeling, thereby enhancing segmentation accuracy. With the U-shaped CNN–Transformer hybrid design, TransUNet demonstrates superior segmentation performance compared to purely convolutional or transformer-based methods.

Given these advantages, we hypothesize that TransUNet’s robust feature extraction capabilities, specifically its proficiency in simultaneously modeling detailed local information and global dependencies, can extend effectively beyond segmentation tasks. In this work, we show that TransUNet’s architecture is well-suited for sparse-view CBCT reconstruction, where precise and globally aware feature extraction is critical to reconstruct high-quality volumetric images from extremely limited X-ray projection data.

### 3.2. The Architecture of Proposed Trans-CBCT

**Figure 2:**
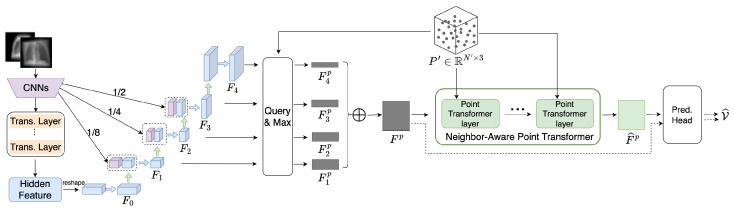
The architecture of Trans-CBCT and Trans^2^-CBCT. Projection images are first encoded using TransUNet to extract multi-scale feature maps. Features from multiple decoder levels are queried and concatenated (⊕) into point features $F^{p}$. Feeding $F^{p}$ directly to the prediction head (dashed arrow) produces the Trans-CBCT model. Alternatively, passing $F^{p}$ through a Point Transformer yields spatially aware features ${\hat{F}}^{p}$, which are then used for final prediction in the Trans^2^-CBCT model.

Following prior works [6,7,8,31], we formulate the CBCT reconstruction as learning a continuous intensity field. Given a 3D spatial domain P and *M* sparse projection images $I=\{I_{1},...,I_{M}\}$, the goal is to learn a continuous function *f* mapping each spatial point p∈P to its attenuation coefficient:

(1) $$\hat{V}=\left\{\hat{v}\left(p\right)=f\left(I,p\right)\;|\;\forall p\in P\right\}.$$

where $\hat{V}$ denotes the reconstructed 3D attenuation coefficient field. To effectively extract meaningful feature representations from limited projections, we employ TransUNet as a feature encoder, shared across all *M* projections. Specifically, TransUNet extracts multi-scale feature maps $\left\{F_{s,1},...,F_{s,M}\right\}$, where each feature map $F_{s,m}\in R^{W_{s}\times H_{s}\times C_{s}}$ corresponds to scale s∈{1,2,3,4} for view *m*, with $W_{s}$, $H_{s}$, and $C_{s}$ denoting the width, height, and number of channels of the feature map at scale *s*, respectively. Note that the four feature levels correspond to decoder scales of TransUNet and are extracted independently for each projection view. As illustrated in Figure 2 (the subscript *m* is omitted in the figure for clarity), we aggregate features from multiple stages of the decoder rather than relying solely on the final-level features. This multi-scale fusion strategy ensures that our model simultaneously captures both low-level spatial features and high-level semantic context.

During training, for a given point p∈P, we first project *p* onto each of the *M* image planes using the corresponding projection function $\pi_{m}$. We then compute view-specific features by performing bilinear interpolation on the feature maps at each scale:

(2) $$F_{s,m}^{p}\left(p\right)=\mathrm{Interp}\left(F_{s,m},\pi_{m}\left(p\right)\right)\in R^{C_{s}}.$$

where Interp(·) is the bilinear interpolation operator.

Next, we fuse these view-specific features across all *M* projections using max-pooling:

(3) $$F_{s}^{p}\left(p\right)=\max\left(\left\{F_{s,1}^{p}\left(p\right),F_{s,2}^{p}\left(p\right),...,F_{s,M}^{p}\left(p\right)\right\}\right)\in R^{C_{s}}.$$

Finally, we concatenate (⊕) the fused features across different scales, s∈{1,2,3,4}, to produce a rich set of multi-scale features for point *p*:

(4) $$F^{p}\left(p\right)=F_{1}^{p}\left(p\right)⊕F_{2}^{p}\left(p\right)⊕F_{3}^{p}\left(p\right)⊕F_{4}^{p}\left(p\right)\in R^{256+128+64+16}$$

After obtaining the multi-scale features $F^{p}$, we directly feed $F^{p}$ into the prediction head (the dashed line in Figure 2) to produce the final reconstruction, which constitutes the Trans-CBCT model.

### 3.3. The Architecture of Proposed Trans^2^-CBCT

We enhance our Trans-CBCT framework by incorporating our neighbor-aware Point Transformer module, resulting in our full framework, referred to as the Trans^2^-CBCT.

**Neighbor-Aware Point Transformer Layer.** While view-specific features are essential for reconstructing the 3D volume from X-ray projection images, enforcing spatial consistency among voxels is equally important for producing artifact-suppressed and anatomically plausible reconstructions. To promote local smoothness and geometric coherence, we introduce a neighbor-aware Point Transformer module.

**Figure 3:**
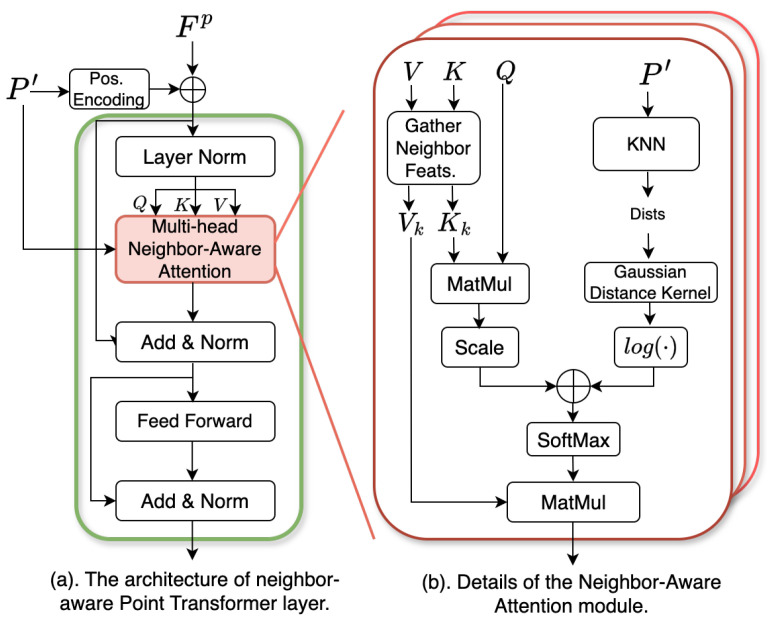
The architecture of neighbor-aware Point Transformer layer. (**a**) Each sampled 3D point is embedded with positional encoding and processed through transformer layers; (**b**) multi-head neighbor-aware attention is restricted to *k*-nearest neighbors, with attention scores guided by both feature similarity and a Gaussian-weighted distance bias, improving local smoothness and geometric consistency.

Rather than processing every 3D point individually, we randomly sample $N^{\prime }$ points from the 3D space P, yielding a set $P^{\prime }\in R^{N^{\prime }\times 3}$. For each sampled point, we query view-specific features using Equations (2)–(4), obtaining a fused multi-scale feature matrix $F^{p}\in R^{N^{\prime }\times 464}$. As illustrated in Figure 3a, the input to the Point Transformer consists of the point coordinates $P^{\prime }$ and their associated features $F^{p}$. To make the transformer aware of 3D geometry, we embed each point $p_{i}\in P^{\prime }$ using a learnable positional encoding:

(5) $$PE\left(p_{i}\right)=\max\left(0,p_{i}W_{1}^{T}+b_{1}\right)W_{2}^{T}+b_{2},$$

where $W_{1}$ and $W_{2}$ are learnable weight matrices, $b_{1}$ and $b_{2}$ are bias terms, and PE(·) is a learnable two-layer MLP-based positional encoding, optimized jointly with the network, rather than a fixed sinusoidal embedding. The positional encoding is added to the multi-scale features $F^{p}$ before feeding them into our neighbor-aware Point Transformer.

**Multi-Head Neighbor-Aware Attention.** Traditional self-attention mechanisms compute pairwise relationships among all input tokens, which can be computationally expensive and may overlook valuable local structures in spatial data. To encourage local smoothness and reduce computational complexity, we propose a multi-head neighbor-aware attention mechanism, illustrated in Figure 3b. For each point $p_{i}\in P^{\prime }$, we identify its *k*-nearest neighbors using a KNN algorithm. From the Euclidean distance between $p_{i}$ and a neighbor $p_{j}$, denoted by $d_{ij}$, we compute a Gaussian-weighted adjacency:

(6) $$w_{ij}=\exp\left(-\frac{d_{ij}^{2}}{2\sigma^{2}}\right),$$

where σ controls the sharpness of the decay. This weighting ensures that nearby points have stronger influence during aggregation.

Each attention head linearly transforms input features into queries *Q*, keys *K*, and values *V*. However, rather than attending to all points, we restrict attention to each point’s local neighborhood. We compute similarity scores as follows:

(7) $${\mathrm{score}}_{ij}=\frac{Q_{i}\cdot K_{j}^{T}}{\sqrt{d_{k}}}+log\left(w_{ij}\right),$$

where $d_{k}$ is the dimension of key vectors. The logarithmic scaling converts the Gaussian weight into an additive distance bias in the attention logits, which improves numerical stability and facilitates integration with dot-product attention. The scores are normalized using a softmax function:

(8) $$\alpha_{ij}=\mathrm{SoftMax}\left({\mathrm{score}}_{ij}\right).$$

The output for each point is then computed by a weighted sum of its neighbors’ value features:

(9) $${\hat{F}}^{p}\left(p_{i}\right)=\sum_{j\in N(i)}\alpha_{ij}V_{j},$$

where N(i) denotes the set of *k*-nearest neighbors of point $p_{i}$. By employing multiple attention heads, we allow the network to capture diverse local patterns. Consequently, our neighbor-aware attention combines both spatial distance and feature similarity to produce robust, context-rich features.

Our neighbor-aware Point Transformer module consists of *l* such layers stacked sequentially. By incorporating geometry-aware positional encodings and neighbor-aware attention, our neighbor-aware Point Transformer promotes local smoothness and spatial consistency in the volumetric representation. This design significantly enhances reconstruction quality by explicitly modeling the spatial structure of the volume, particularly under conditions of extreme projection sparsity.

### 3.4. Model Optimization

For both of our proposed frameworks, Trans-CBCT and Trans^2^-CBCT, the feature representation ${\hat{F}}^{p}\left(p_{i}\right)$ for each point $p_{i}$ is passed through a lightweight attenuation prediction head. This head consists of a sequential stack of Conv1D, BatchNorm, ReLU, Conv1D, and Sigmoid layers, which outputs the predicted attenuation coefficient ${\hat{v}}_{i}$.

Rather than directly reconstructing the entire 3D CT volume, we adopt a point-wise regression strategy. Specifically, we sample a set of points $P^{\prime }$ from the ground-truth CT volume and train the network to predict the corresponding attenuation coefficients. The ground-truth $V=\{v_{1},v_{2},...,v_{N^{\prime }}\}$ for these sample points are obtained by trilinear interpolation from the full-resolution CT volume.

We optimize the model using the mean square error (MSE) loss between the predicted and ground-truth coefficients:

(10) $$L\left(V,\hat{V}\right)=\frac{1}{N^{\prime }}\sum_{i=1}^{N^{\prime }}{\left(v_{i}-{\hat{v}}_{i}\right)}^{2}.$$

## 4. Experiments

### 4.1. Experimental Details

(i) Datasets and Settings

We conduct experiments on two publicly available datasets: LUNA16 [18], which is a chest CT dataset, and ToothFairy [19], which is a dental CBCT dataset. Our implementation adopts the dataset pipeline and projection geometry of DIF-Gaussian [31]. Following [6,31], we split LUNA16 into 738 scans for training, 50 scans for validation, and 100 scans for testing. We also use the same volume resolution and voxel spacing. The chest CT scans are preprocessed so that each volume has shape 256×256×256 with consistent spacing [1.6,1.6,1.6] mm. The ToothFairy dataset is divided as 343 scans for training, 25 scans for validation, and 75 scans for testing. The dental CBCT scans have volume shape of 256×256×256 with spacing [2.1,5.4,5.4] mm. We use digitally reconstructed radiographs (DRRs) to generate the projection images with a resolution of 256×256. The simulated CBCT acquisition adopts a cone-beam geometry with a source-to-origin distance (DSO) of 800 mm and a source-to-detector distance (DSD) of 1200 mm. A total of 50 projection images are uniformly generated over an angular range of $\left[0^{\circ },180^{\circ }\right)$ using a flat-panel detector with pixel size of 2.4×2.4 mm. For sparse-view reconstruction, we uniformly sample M=6,8, or 10 projections with equal angular spacing from the full set.

(ii) Experimental Setup

All the experiments are conducted on an NVIDIA Quadro RTX 6000 (NVIDIA, Santa Clara, CA, USA) with Python 3.8.20 and PyTorch 1.13.0. The model is optimized with the AdamW [46] optimizer with a learning rate of 0.001 and weight decay of $1\times 10^{-6}$. The model is trained for 400 epochs with batch size of 2 for both datasets. $N^{\prime }=10,000$ points are sampled as part of the input. Voxel intensities are clipped to the range of [−1000, 1024] HU and linearly normalized to [0, 1]. To balance computational efficiency and reconstruction accuracy, we use l=2 Point Transformer layers and set k=3 for KNN algorithm in neighbor-aware attention. We use the peak signal-to-noise ratio (PSNR) [16] and structural similarity index measure (SSIM) [17] as the performance metrics. Higher values mean better reconstruction results.

(iii) Baselines

Following [31], we compare our model with three types of baselines, namely self-supervised methods, denoising-based data-driven methods, and implicit neural representation (INR)-based data-driven methods. Self-supervised methods, which require no external CT scans but only projection images, include FDK [20], SART [2], NAF [8], and NeRP [30]. Denoising-based methods include FBPConvNet [28], FreeSeed [47] and BBDM [48]. INR-based methods include PixelNeRF [29], DIF-Net [6], DIF-Gaussian [31], and C^2^RV [7]. For DIF-Gaussian* [31], in Table 1 and Table 2 we reproduced the experiments given the original authors’ implementation. The remaining results for other methods (except C^2^RV) are from DIF-Gaussian [31]. The results for C^2^RV are from [7].

### 4.2. Quantitative Results

We evaluate all methods at a fixed resolution of $256^{3}$ using 6, 8, or 10 projection views. As detailed in Section 4.1, the results for the baselines other than DIF-Gaussian* [31] are cited from previously published papers, since not all baseline implementations are publicly available. Nevertheless, all methods are evaluated on the same dataset pipeline, projection geometry, and evaluation metrics for a fair comparison. The results for LUNA16 and ToothFairy datasets are summarized in Table 1 and Table 2, respectively. As shown in these tables, self-supervised reconstructions (FDK, SART, and NeRP) yield the lowest fidelity, caused by lacking learned priors to suppress artifacts. Denoising networks (FBPConvNet; BBDM) improve results by applying data-driven post-processing. Implicit neural representation-based methods (PixelNeRF, DIF-Net, DIF-Gaussian, and C^2^RV) further advance performance by modeling the continuous attenuation field and incorporating learned priors. Notably, on LUNA16 with just six views (Table 1), our Trans-CBCT framework already exceeds the best baseline (INR-based C^2^RV) by 1.17 dB in PSNR and $1.63\times 10^{-2}$ in SSIM, demonstrating the enhanced representational capacity of TransUNet’s hierarchical feature maps. Building on this, Trans^2^-CBCT, with its Point Transformer and neighbor-aware attention, delivers additional improvement over Trans-CBCT under the same six-view setting and provides an increase of 1.8 dB in PSNR and $2.8\times 10^{-2}$ in SSIM over the best baseline (INR-based C^2^RV).

A similar trend holds on the ToothFairy dataset, as shown in Table 2. Trans^2^-CBCT outperforms all baselines in five of the six measurements. It gives the second best SSIM value ($92.16\times 10^{-2}$) for one of the settings, which is very comparable to the best number ($92.81\times 10^{-2}$). Both Trans-CBCT and Trans^2^-CBCT consistently outperform all baselines across all settings in terms of PSNR, providing the best and second-best performances. Under the six-view setting, Trans-CBCT surpasses the best performing baseline C^2^RV by 2.19 dB in PSNR. Trans^2^-CBCT further improves performance and surpasses C^2^RV by 2.75 dB in PSNR. These results confirm that explicit 3D spatial reasoning and local smoothness regularization are pivotal for high-fidelity sparse-view CBCT reconstruction.

### 4.3. Qualitative Results

**Figure 4:**
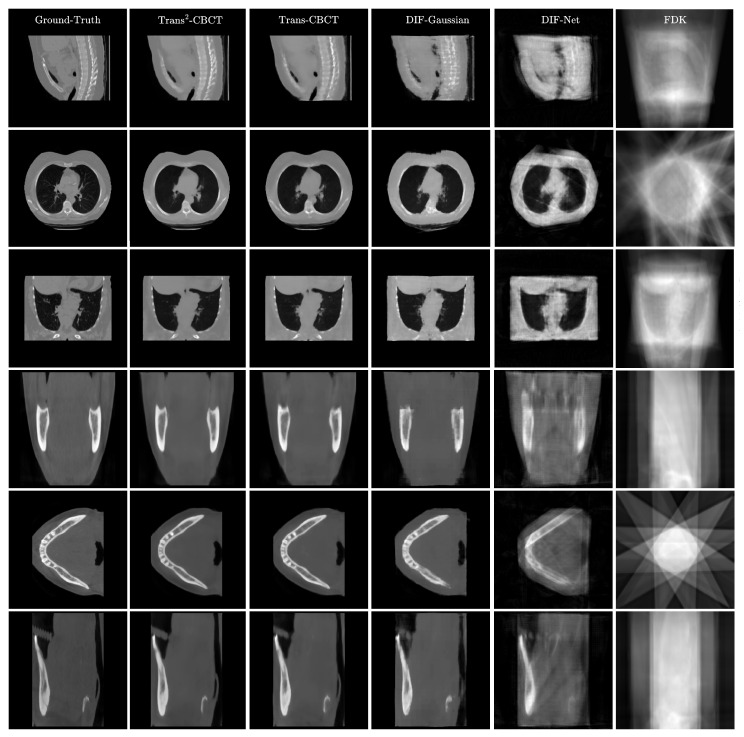
Visualization of 6-view reconstruction results for different methods. Top three rows: sagittal, axial, and coronal slices from the LUNA16 dataset. Bottom three rows: corresponding slices from the ToothFairy dataset.

**Figure 5:**
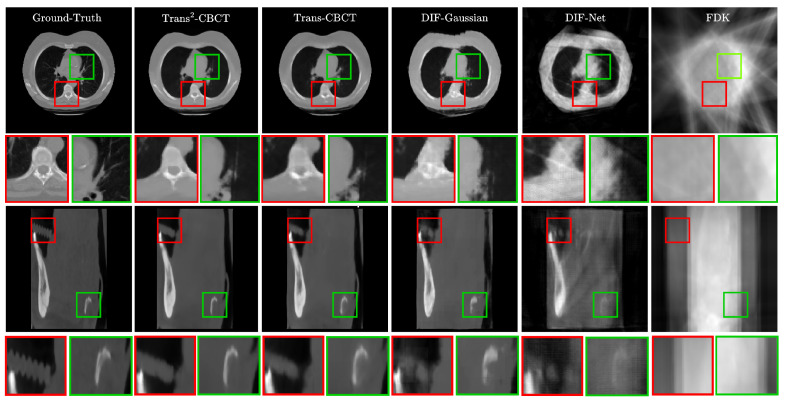
Zoomed-in comparison of 6-view reconstruction results for different methods. The highlighted regions (red boxes and green boxes) are zoomed in, showing more details. Top row: Axial slices from the LUNA16 dataset. Bottom row: corresponding slices from the ToothFairy dataset.

We provide a qualitative comparison of six-view reconstruction results across different methods in Figure 4, with zoomed-in comparisons shown in Figure 5. Overall, the differences among methods highlight the challenges of extremely sparse-view CBCT reconstruction and the advantages of our proposed approaches.

The FDK algorithm [20] struggles under such sparse-view settings and cannot reconstruct meaningful anatomical structures, resulting in highly blurred images dominated by streaking and shading artifacts.

DIF-Net [6] shows clear improvements over FDK. It successfully recovers the overall anatomical contours, such as the lung boundaries and the dental arch shapes. However, it still suffers from noticeable limitations: many fine structures are lost, and strong streak artifacts remain present throughout the volume, particularly around high-contrast edges. This suggests that DIF-Net has difficulty capturing intricate details from extremely limited projections.

DIF-Gaussian [31] further improves upon DIF-Net by introducing a more expressive Gaussian parameterization. As shown in the fourth column of Figure 4, DIF-Gaussian produces cleaner reconstructions with sharper boundaries and better preservation of internal structures. Nevertheless, some blurring and smoothing effects are still evident, especially in regions requiring high-frequency detail recovery, such as small vessel structures in chest images or fine bone textures in dental scans.

As can be seen, our proposed Trans-CBCT and Trans^2^-CBCT models provide the best visual quality. Specifically, Trans-CBCT generates highly detailed reconstructions with sharp tissue interfaces and significantly reduced streak artifacts compared to previous baselines. Building on this, Trans^2^-CBCT further refines local anatomical details, offering even clearer internal structures and more consistent intensity distributions across slices.

The performance gains can be attributed to the combination of multi-scale feature extraction by TransUNet and spatially aware local refinement by the neighbor-aware Point Transformer. Together, they enable our model to better integrate sparse-view information while preserving both global structure and local details. These results clearly demonstrate the effectiveness of our framework for overcoming the severe information loss caused by extremely sparse projection sampling.

### 4.4. Efficiency Analysis

We evaluate the computational efficiency of our proposed models, Trans-CBCT and Trans^2^-CBCT, against two INR-based baselines, DIF-Net and DIF-Gaussian, with results summarized in Table 3. In terms of trainable parameters, our models are indeed more complex due to the use of TransUNet, which incorporates Transformer layers known to be parameter-intensive. However, this increased capacity enables stronger feature extraction and better reconstruction performance from sparse-view inputs. Notably, despite the larger model size, Trans-CBCT maintains competitive runtime. It requires only marginally more time (1.5 s) than DIF-Net and outperforms DIF-Gaussian in inference speed by running 1.9 s faster. This suggests that our model architecture is not only effective but also efficiently designed for practical use, particularly given the improved reconstruction quality it delivers. Trans^2^-CBCT takes 53.7 s to reconstruct one case. This overhead stems mainly from the KNN-based neighbor search required by the neighbor-aware attention module, which computes the *k*-nearest neighbors for each of 10,000 sampled points. Although this step improves local smoothness and spatial consistency, it introduces additional computational cost. We identify this as a key area for optimization; implementing a more efficient or approximate KNN algorithm could significantly reduce runtime without sacrificing quality.

Overall, Trans-CBCT offers a compelling tradeoff between reconstruction fidelity and inference efficiency. It demonstrates that Transformer-based architectures, when carefully integrated, can outperform traditional convolutional or MLP-based models not only in accuracy but also in practical runtime.

### 4.5. Ablation Studies

(i) Analysis of the Number of Sampled Points $N^{\prime }$

Table 4 shows the effect of the number of sampled points, $N^{\prime }$, on the performance. When using $N^{\prime }=5000$ points, our Trans^2^-CBCT framework provides relatively lower PSNR and SSIM values. Increasing $N^{\prime }$ to 10,000 significantly boosts performance on both datasets, indicating that denser sampling helps the model learn a more accurate and smoother attenuation field. However, further increasing $N^{\prime }$ to 20,000 does not lead to continued improvement. On the contrary, performance slightly degrades compared to 10,000, suggesting that too many sampled points may introduce redundant information, cause overfitting to sampled training points, or slow down convergence. These results demonstrate that setting $N^{\prime }=$ 10,000 reaches the best balance between reconstruction accuracy and training efficiency and is therefore adopted as the default configuration in our experiments.

(ii) Analysis of the Number of Neighbors

We conduct an ablation study on the number of *k*-nearest neighbors used in the neighbor-aware attention module, with results summarized in Table 5. The experiments are performed on both the LUNA16 and ToothFairy datasets. Using k=3 achieves the highest reconstruction quality, which indicates that a small number of neighbors is sufficient to capture local geometric structure without over-smoothing fine details. As *k* increases to 6, 9, and 15, we observe a slight decline in both the PSNR and SSIM values. These results suggest that involving too many neighbors in the attention operation causes excessive feature smoothing and blurs fine anatomical boundaries. In addition to better reconstruction quality, using a smaller *k* also improves computation efficiency. A smaller number of neighbors reduces the complexity of the KNN search and the subsequent attention computation, leading to faster inference and lower memory usage during both training and testing. Therefore, setting k=3 offers the best trade-off between reconstruction fidelity and computational efficiency and is adopted as the default configuration in our final Trans^2^-CBCT model.

(iii) Multi-Level Features

We conduct an ablation study on different feature aggregation strategies, as summarized in Table 6. Using only feature $F_{4}$ leads to the lowest reconstruction performance, while adding $F_{3}$ improves results, and adding another intermediate feature $F_{2}$ further enhances performance. This indicates that integrating mid-level semantic information helps refine fine anatomical structures. The full multi-level feature concatenation, which incorporates $F_{1}$, $F_{2}$, $F_{3}$, and $F_{4}$, yields the best performance. These results demonstrate that aggregating multi-scale features across multiple decoder stages improves reconstruction fidelity compared to using only deep or partial features. This validates the effectiveness of our multi-level feature aggregation design in capturing both fine details and global structure for sparse-view CBCT reconstruction.

(iv) Analysis of the Image Encoder

We conduct an ablation study to evaluate the contribution of the image encoder in Trans^2^-CBCT. We replace it with a standard UNet encoder while keeping the neighbor-aware Point Transformer unchanged. The results summarized in Table 7 show that replacing our encoder with a standard UNet leads to a 0.45 dB decrease in PSNR and $1.5\times 10^{-2}$ decrease in SSIM on LUNA16 with six views, demonstrating that our encoder captures richer local and global 2D features compared to the standard UNet.

## 5. Conclusions

In this paper, we have proposed Trans-CBCT and Trans^2^-CBCT, two transformer-based frameworks, for reconstructing high-quality CBCT volumes from an extremely sparse number of X-ray projection views. Trans-CBCT employs TransUNet to extract multi-scale, long-range-aware features from each projection, enabling effective artifact suppression and robust 3D feature querying. To further enhance 3D spatial consistency, Trans^2^-CBCT extends this design by integrating a Point Transformer equipped with learnable 3D positional encodings and a neighbor-aware attention mechanism. This component enforces local smoothness and preserves anatomical details, particularly in highly undersampled settings. Extensive experiments on both the LUNA16 and ToothFairy datasets demonstrate the strengths of our models. On the LUNA16 dataset, they consistently outperform analytic and learning-based baselines in terms of both the PSNR and SSIM. On the ToothFairy dataset, Trans^2^-CBCT outperforms all baselines in five of the six measurements, and both Trans-CBCT and Trans^2^-CBCT outperform all baselines across all settings in terms of PSNR. Notably, Trans-CBCT achieves a strong balance between reconstruction quality and efficiency, while Trans^2^-CBCT pushes the state of the art in fidelity. In summary, our work demonstrates the effectiveness of combining advanced 2D feature extraction with 3D geometric reasoning for CBCT reconstruction. We believe that Trans-CBCT and Trans^2^-CBCT provide a strong foundation for future research on transformer-based volumetric imaging in sparse-view scenarios, with potential applications in low-dose medical imaging and mobile diagnostic systems. It is important to note that while PSNR and SSIM provide quantitative comparison with prior work, they do not capture diagnostic utility. The diagnostic value of the reconstructed images has not been clinically validated, and a radiological evaluation or a reader study is an essential step before any clinical deployment.

## Figures and Tables

**Table 1:** Quantitative comparison of baselines on the LUNA16 dataset from 6, 8, or 10 projection views. Higher values of PSNR (dB) and SSIM ($\times 10^{-2}$) indicate better reconstruction quality. Our models Trans^2^-CBCT and Trans-CBCT achieve the best and second-best performance, shown as **bold** and underlined, respectively. The results for DIF-Gaussian* are reproduced, while the other baseline results are cited from the literature.

Method	Type	LUNA16					
		6-View		8-View		10-View	
		PSNR	SSIM	PSNR	SSIM	PSNR	SSIM
FDK [20]	Analytical	15.34	35.78	16.58	37.89	17.40	39.85
SART [2]	Iterative	19.70	64.36	20.06	67.80	20.23	70.23
NAF [8]	Self-supervised: INR-based	18.76	54.16	20.51	60.84	22.17	62.22
NeRP [30]		23.55	74.46	25.83	80.67	26.12	81.30
FBPConvNet [28]	Supervised: Denoising	24.38	77.57	24.87	78.86	25.90	80.03
FreeSeed [47]		25.59	77.36	26.86	78.92	27.23	79.25
BBDM [48]		24.78	77.03	25.81	78.06	26.35	79.38
PixelNeRF [29]	Fully-supervised: INR-based	24.66	78.68	25.04	80.57	25.39	82.13
DIF-Net [6]		25.55	84.40	26.09	85.07	26.67	86.09
DIF-Gaussian* [31]		27.71	84.87	28.71	86.43	29.07	87.31
C^2^RV [7]		29.23	87.47	29.95	88.46	30.70	89.16
Trans-CBCT (ours)		30.40	89.10	31.12	90.07	31.82	90.88
Trans^2^-CBCT (ours)		**31.03**	**90.27**	**31.70**	**91.05**	**32.44**	**91.88**

**Table 2:** Quantitative comparison of baselines on the ToothFairy dataset with 6, 8, or 10 projection views. Higher values of PSNR (dB) and SSIM ($\times 10^{-2}$) indicate better reconstruction quality. Trans^2^-CBCT outperforms all baselines in five of the six measurements. Both Trans-CBCT and Trans^2^-CBCT consistently outperform all baselines across all settings in terms of PSNR, providing the best and second-best performances, shown as **bold** and underlined, respectively. The results for DIF-Gaussian* are reproduced, while the other baseline results are cited from the literature.

Method	Type	ToothFairy					
		6-View		8-View		10-View	
		PSNR	SSIM	PSNR	SSIM	PSNR	SSIM
FDK [20]	Analytical	17.07	39.90	18.42	43.29	19.58	47.21
SART [2]	Iterative	20.04	64.98	21.92	67.86	22.82	71.53
NAF [8]	Self-supervised: INR-based	20.58	63.52	22.39	67.24	23.83	72.52
NeRP [30]		21.77	72.06	24.18	78.83	25.99	82.08
FBPConvNet [28]	Supervised: Denoising	27.22	79.33	27.72	81.90	28.13	83.51
FreeSeed [47]		26.35	78.98	27.08	81.38	27.63	84.40
BBDM [48]		26.29	78.57	27.28	80.33	28.00	83.96
PixelNeRF [29]	Fully-supervised: INR-based	24.85	80.91	25.37	82.11	25.90	83.25
DIF-Net [6]		25.78	83.62	26.29	84.81	26.90	86.42
DIF-Gaussian* [31]		28.81	87.06	29.83	88.67	29.07	85.17
C^2^RV [7]		28.85	**92.81**	29.30	93.03	30.04	93.38
Trans-CBCT (ours)		31.04	91.09	32.11	92.32	32.55	92.72
Trans^2^-CBCT (ours)		**31.60**	92.16	**32.71**	**93.27**	**33.63**	**93.96**

**Table 3:** Efficiency analysis comparing trainable parameters and inference time of different methods.

Method	Trainable Param. (MB)	Inference Time (s)
DIF-Net	31.1	8.4
DIF-Gaussian	31.7	11.8
Trans-CBCT	105.4	9.9
Trans^2^-CBCT	108.1	53.7

**Table 4:** Ablation study on the number of sampled points $N^{\prime }$ during training.

$N^{\prime }$	LUNA16		ToothFairy	
	PSNR	SSIM	PSNR	SSIM
5000	28.49	87.13	31.15	91.42
10,000	31.03	90.27	31.6	92.16
20,000	30.40	89.71	31.27	91.62

**Table 5:** Ablation study on the number of *k*-nearest neighbors in the Neighbor-Aware Attention module.

	LUNA16		ToothFairy	
	PSNR	SSIM	PSNR	SSIM
k = 3	31.03	90.27	31.60	92.16
k = 6	30.23	89.37	31.50	91.95
k = 9	29.91	89.04	31.40	91.75
k = 15	29.76	88.83	31.09	91.20

**Table 6:** Experiments on different feature aggregation strategies. Multi-level feature concatenation significantly improves reconstruction performance.

Feature Strategy	Trans-CBCT		Trans^2^-CBCT	
	PSNR	SSIM	PSNR	SSIM
F4	26.82	80.46	28.28	85.32
F4; F3	28.05	82.61	29.60	87.81
F4; F3; F2	28.48	83.69	30.18	88.89
Full multi-level	30.40	89.10	31.03	90.27

**Table 7:** Experiments on Trans^2^-CBCT with a standard UNet encoder.

Method	PSNR (dB)	SSIM ($\times 10^{-2}$)
UNet encoder	30.58	88.77
Trans^2^-CBCT	31.03	90.27

## Data Availability

The datasets used in this study (LUNA16 [18] and ToothFairy [19]) are publicly available from their respective authors.
